# How to evaluate the implementation of complex health programmes in low-income settings: the approach of the Gavi Full Country Evaluations

**DOI:** 10.1093/heapol/czaa127

**Published:** 2020-11-06

**Authors:** Caroline Soi, Jessica C Shearer, Ashwin Budden, Emily Carnahan, Nicole Salisbury, Gilbert Asiimwe, Baltazar Chilundo, Haribondhu Sarma, Sarah Gimbel, Moses Simuyemba, Jasim Uddin, Felix Masiye, Moses Kamya, Dai Hozumi, Julie K Rajaratnam, Stephen S Lim

**Affiliations:** 1 Health Alliance International, Mozambique and University of Washington, USA; 2 PATH; 3 D’Eva Consulting; 4 Gavi; 5 Universidade Eduardo Mondlane; 6 icddr,b; 7 Health Alliance International, University of Washington; 8 University of Zambia; 9 Infectious Diseases Research Collaboration; 10 IntraHealth; 11 Institute for Health Metrics and Evaluation, University of Washington

**Keywords:** Evaluation, implementation research, immunization, health systems

## Abstract

Vaccination, like most other public health services, relies on a complex package of intervention components, functioning systems and committed actors to achieve universal coverage. Despite significant investment in immunization programmes, national coverage trends have slowed and equity gaps have grown. This paper describes the design and implementation of the Gavi Full Country Evaluations, a multi-country, prospective, mixed-methods approach whose goal was to monitor and evaluate processes, inputs, outputs and outcomes of immunization programmes in Bangladesh, Mozambique, Uganda and Zambia. We implemented the Full Country Evaluations from 2013 to 2018 with the goal of identifying the drivers of immunization programme improvement to support programme implementation and increase equitable immunization coverage. The framework supported methodological and paradigmatic flexibility to respond to a broad range of evaluation and implementation research questions at global, national and cross-country levels, but was primarily underpinned by a focus on evaluating processes and identifying the root causes of implementation breakdowns. Process evaluation was driven by theories of change for each Gavi funding stream (e.g. Health Systems Strengthening) or activity, ranging from global policy development to district-level programme implementation. Mixing of methods increased in relevance and rigour over time as we learned to build multiple methods into increasingly tailored evaluation questions. Evaluation teams in country-based research institutes increasingly strengthened their level of embeddedness with immunization programmes as the emphasis shifted over time to focus more heavily on the use of findings for programme learning and adaptation. Based on our experiences implementing this approach, we recommend it for the evaluation of other complex interventions, health programmes or development assistance.

KEY MESSAGES

- Health impact is lost due to the sub-optimal implementation of health programmes, activities and interventions.
- Careful evaluation of the implementation of health programmes can illuminate the ‘black box’ of implementation and identify solvable bottlenecks, even for the most complex programmes. These types of evaluations should be driven by theory, mix methods and involve multi-disciplinary teams.
- The Gavi Full Country Evaluations, a multi-country, mixed-methods, prospective evaluation adapted over time to become more responsive to stakeholder needs and to emphasize learning and use of findings. Its design and evolution provides a blueprint for evaluators of other complex health programmes.

## Introduction

Vaccination is one of the most cost-effective public health interventions (Ozawa *et al.*, 2016). Yet, an estimated 19.4 million children remain un- or under-immunized  (World Health Organization, 2019). Immunization programmes are highly complex, particularly as they shift beyond ‘business as usual’ approaches to reaching the majority of children, to reaching all children by overcoming entrenched obstacles that constrain universal immunization coverage. Improving the performance of immunization programmes requires a clear understanding of the bottlenecks to progress and strategies to address them; in other words, vaccine programmes must be rigorously evaluated to learn and improve. Despite their potential for health impact, few vaccine programmes have been rigorously evaluated with a view towards systems complexity, real-time learning and continuous improvements in programme design and delivery (Paina and Peters, 2012). Immunization programmes present a testing ground for evaluation and implementation research (IR) approaches to achieve universal health coverage. The objective of this paper is to describe the design and implementation of this prospective, mixed-methods programme evaluation and to summarize best practices for IR and evaluation based on our experiences, which evolved over time. While we primarily use the term ‘evaluation’ in this paper, we identify how our own definition of ‘evaluation’ shifted over time; components of the larger evaluation may be more commonly thought of as IR.

### The state of programme evaluation and IR

Recent years have seen a growth in interest—and application—of methods, approaches and tools from developmental evaluation (Patton, 1994) and IR to similar questions. While the communities of evaluation and IR are often distinct, there is growing convergence of these fields due to a shared objective: to improve the health impact of increasingly complex interventions or programmes. IR and related disciplines (e.g. dissemination research, knowledge translation), have made important contributions to highlighting the ‘know-do’ gap; even the most effective interventions will fail without effective implementation (Damschroder *et al.*, 2009). In parallel, evaluators stemming largely from the sub-disciplines of developmental and realist evaluation began emphasizing the need for evaluating processes in addition to outcomes, and using theory to inform approached to understand why and how something works (Pawson and Tilley, 1997; Campbell, 2000; Craig *et al.*, 2008; Moore *et al.*, 2015). This latter emphasis is shared by health systems and policy researchers who have been calling for a greater use of social science theory (Gilson *et al.*, 2011). These shifts in goals require new flexible, forward-thinking and theory-driven approaches to investigate multi-faceted and dynamic aspects of programme implementation (Rogers, 2008; Gerrits and Verweij, 2015; Nieminen and Hyytinen, 2015). The increasing use of methods from systems thinking and complexity science disciplines has contributed to the evaluation of complex interventions in complex systems (Savigny *et al.*, 2009; Paina and Peters, 2012; Masset and White, 2019). Recently, the field of programme evaluation has accepted the importance of designing evaluation to support learning and adaptation (Keith *et al.*, 2017;USAID, 2017;  Andrews *et al*., 2016), a shift from the evaluation field’s historical focus on methods to reduce sources of bias and maintain independence.

### Background

Gavi, the Vaccine Alliance (hereafter referred to as ‘Gavi’) invests not only in vaccines and supplies for immunization programmes, but also in strengthening health and immunization systems. Since its inception in 2000, Gavi has disbursed $13.4 billion  (Gavi, 2019)  and was an early adopter of the global health partnership model of governance and implementation (Brinkerhoff, 2002; Kamya *et al.*, 2016). Despite commissioning traditional retrospective evaluations of its investments, Gavi had limited evidence on how, or whether, its investments contributed to immunization programme improvement. In this context, Gavi commissioned the Full Country Evaluations (FCE) in Bangladesh, Mozambique, Uganda and Zambia. The evaluation objectives of the two phases were:

- Phase 1 (2013–16): to understand and quantify the barriers to and drivers of immunization programme improvement, including Gavi’s contributions.
- Phase 2 (2017–18): to evaluate the new policies, programmes and processes implemented by the Gavi strategy for the 2016–2020 period with a focus on identifying the drivers of equitable coverage and Gavi’s contribution to observed changes.

## Design of the overarching evaluation framework

The FCE was a multi-country, prospective, mixed-methods evaluation. Embedded in the overarching objectives were multiple sub-evaluation and IR activities designed to answer over 40 specific evaluation questions (EQs) (see Web Appendix 1). Gavi initially posed 22 questions in the FCE Request for Proposals and new questions were added over time, increasingly by country stakeholders. We wrote EQs to be increasingly targeted in scope and analytic in nature as the broad features of how countries implemented Gavi support became clearer.

**Figure 1:**
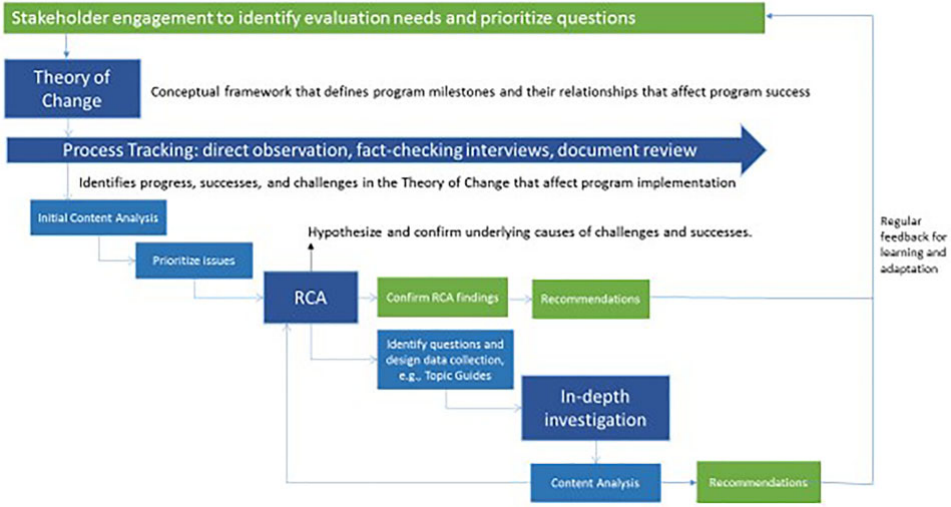
Evaluation approach

Table 1 summarizes design elements of the FCE. From the outset, a *prospective* design with an emphasis on process evaluation (Moore *et al.*, 2015) was considered essential for illuminating what global and national stakeholders perceived as the ‘black box’ of country-level immunization programme implementation. Neither Gavi, partners, nor governments had a good sense of how national vaccine policy decisions were made, what happened to Gavi funding—particularly health system strengthening (HSS) funding—once it was disbursed, or how even highly replicated processes of introducing new vaccines were implemented in reality. In line with guidance on evaluating complex evaluations that was available at FCE’s inception, we used process evaluation to understand ‘why an intervention fails or has unexpected consequences, or why a successful intervention works and how it can be optimized’ (Craig *et al.*, 2008). We implemented process evaluation prospectively to enable real-time feedback for learning and action, to observe unintended consequences, to more easily observe and test causal mechanisms. We initiated prospective observation as early as possible in the results chain, believing that policy and operational decision-making would have important consequences on programme implementation (Figure 1).

**Table 1:** Design elements of a prospective, mixed-methods, comprehensive, process-focused evaluation

Element	The FCE approach
Overarching objectives	Phase 1 (2013–16): to understand and quantify the barriers to and drivers of immunization programme improvement, including Gavi’s contributions Phase 2 (2017–18): to evaluate the new policies, programmes and processes implemented by the Gavi strategy for the 2016–2020 period with a focus on identifying the drivers of equitable coverage and Gavi’s contribution to observed changes
Time frame	Prospective
Knowledge paradigm and disciplines	Mixed paradigms (positivist and relativist) and multidisciplinary [implementation science, evaluation, epidemiology, biostatistics, public health, social science (policy science, organizational science, health services research), economics]
Methods	Multiple, mixed methods driven by the EQ or phenomenon of interest
Scope	All aspects of the results chain and all levels of intervention (from global policies and process to national and subnational implementation) related to Gavi investments and national immunization programmes
Data sources	Multiple data sources, often triangulated to answer a single question. An emphasis on fit-for-purpose data collection tailored to specific EQs
Analytic approach	Multiple analytic approaches based on fit with EQ and evidence needs. Methods and analytic approaches were increasingly purposively mixed over time as we improved question framing to allow mixing.
Generalizability and comparability	Use of ToCs and conceptual frameworks support comparability across multiple countries
Evaluators and participants	Evaluators were staff of national universities or research institutions, and global research institutions at global level. Evaluators shifted from independent arms-length evaluators to, in some cases, participant-observers of EPI processes and decisions. Global and national immunization stakeholders participated in the design of the evaluation and selection of EQs.
Dissemination and feedback loops	Over time, the consortium implemented regular dissemination and feedback to key national-level stakeholders (e.g. active participation during EPI meetings, quarterly policy briefs), including specific and targeted recommendations
Governance	Commissioned by the Evaluation Advisory Committee, a sub-committee of the Gavi Board composed of independent evaluation advisors. Funded by Gavi, and managed by the evaluation team at the Gavi Secretariat.

Gavi and the evaluation consortium recognized the need for multiple, *mixed methods* and multiple disciplinary perspectives if we were to answer any of the possible range of EQs. The consortium was composed of global partners with skills in quantitative and qualitative methods, and country-based, multi-disciplinary evaluation partners. A key lesson learned from our consortium was the benefit of physically co-locating team members of various disciplinary and methods backgrounds—without this we found that actual mixing of methods and paradigms was unlikely to happen. Similarly, we learned over time that it was helpful to pose EQs in a way that encouraged trans-paradigm and mixed-methods data collection and analysis, relying often on ‘whether, why and how does X outcome occur’-type questions. Early in the evaluation our approach to mixed methods often relied on using data sources sequentially to explain the other, as illustrated in Table 2. The limitation of this approach was that it did not leverage the strengths of the prospective approach, often resulting in additional questions from one data source that could no longer be answered retrospectively from the other. Without intentional design incorporating multiple methods and data sources, mixing was often sub-optimally used (Carnahan *et al*., 2020).

**Table 2:** Sequential vs simultaneous mixing

Sequential mixing of methods	Simultaneous mixing of methods
We frequently used quantitative data sources to identify low coverage or sub-optimal routinization of a new vaccine and used qualitative methods to identify the root causes. In Mozambique, e.g. we observed low coverage of measles second dose (MSD) through examination of routine HMIS data. The evaluation team in Mozambique developed hypotheses to explain low coverage and tested them through in-depth interviews with national and sub-national EPI stakeholders.	We used multiple methods simultaneously to answer the FCE EQ related to partnership (‘What is the effectiveness, efficiency, and country ownership of national immunization partnerships and their contribution program performance’.). We employed social network mapping and qualitative interviews at the same time to develop a holistic understanding of how the structure of immunization partnerships influenced their performance and observed outcomes (Kamya *et al.*, 2016).

Evaluating a complex programme required multiple knowledge paradigms and world views (Gilson *et al.*, 2011). The FCE employed positivist and relativist paradigms but tended towards relativism for many research questions. To achieve this standard of theory-driven evaluation we involved an increasingly diverse team or network of evaluators and researchers, with a growing emphasis on the importance of social science theory and relativist question-asking to inform our work over time.

The evaluation was designed to support feedback and adaptation (Patton, 1994), not only through the prospective aspect but also through engagement of potential users to co-design EQs, and quasi-embeddedness of evaluators in national EPI programmes. In terms of engagement, while Gavi developed the original FCE Request for Proposals with inputs from stakeholders, over time we increasingly used a range of formal and informal approaches to engaging and co-designing with stakeholders and potential users, including the Gavi Secretariat and Alliance partners and national EPI managers and country-based Alliance partners. The evaluation consortium’s frame shifted over time to the evidence needs of national EPI programmes and their stakeholders, and while national and sub-national needs were typically aligned with global-level evaluation priorities, there were instances where the evaluation priorities of the Gavi Secretariat M&E team or Gavi’s Evaluation Advisory Committee (EAC) differed from those of country-based stakeholders, requiring careful management of agendas and priorities across many complex stakeholders. We sought, where possible, to respond to timely and local stakeholder questions to engender trust in the team and findings and increase their likelihood of use.

Evaluation teams frequently attended EPI meetings and spoke regularly to key programme personnel as part of the evaluation, enabling rich contextual observations necessary for evaluation of complex interventions (Moore *et al.*, 2015). In Uganda, in particular, the evaluation team’s near-daily engagement and strong relationships contributed to the EPI programme and partners viewing them as one of the partners to contribute insights and ideas. Early on in the FCE, the consortium debated the boundaries of independence as evaluators, vs ethical obligations to contribute to programme improvement. In the years since that time the larger discipline has become more confident not only in its role in adaptation and learning, but also in trusting the strength of process evaluation to track evaluator reflexivity (Koch and Harrington, 1998;  Jacobson *et al*., 2009).

Musing on *design*: We advocate for a prospective approach, but multiple methods must be designed and implemented concurrently and by a coordinated multi-disciplinary team. Feedback loops are likely to be stronger if evaluators are more embedded in the programme.

### Scope

**Figure 2:**
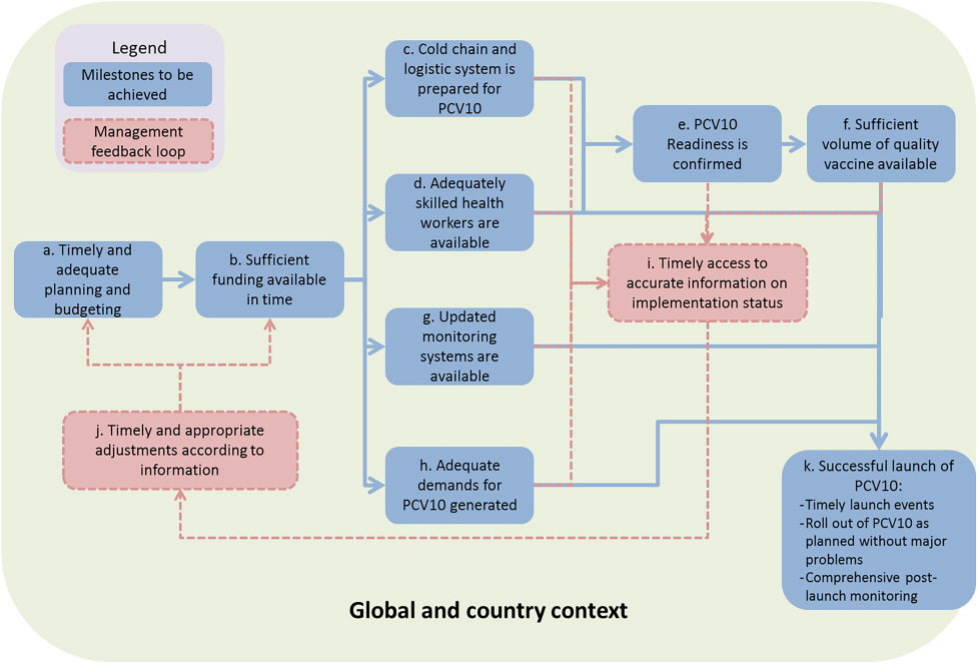
Example ToC (process of introducing pneumococcal conjugate vaccine)

The FCE covered the full M&E results chain (inputs, activities, outputs, outcomes and impact) for all Gavi support streams. A support stream refers to Gavi’s specific investment areas, including New Vaccine Support, Health Systems Strengthening (Health Systems and Immunization Systems Strengthening, since 2016), and Supplementary Immunization Activities (e.g. vaccination campaigns).^1^ Through process evaluation we comprehensively assessed the inputs, activities and outputs around each support stream, across different phases of action: (1) global policy, communication and technical assistance related to the stream, (2) countries’ decision to apply for support, (3) application, (4) planning and (5) implementation. We developed detailed theories of change (ToCs) to structure tracking and measurement of each support stream (Figure 2). ToCs identified intended causal pathways and related indicators . The ToCs were informed by evidence and tacit knowledge related to the processes, yet we acknowledged they overly simplified reality. The process evaluation was designed to incorporate elements of context and complexity through further data collection and analysis initiated when evaluators observed a deviation from the ToC . To support this flexibility, Gavi set aside funding for unanticipated data collection that had not been anticipated during the annual design process.

**Figure 3:**
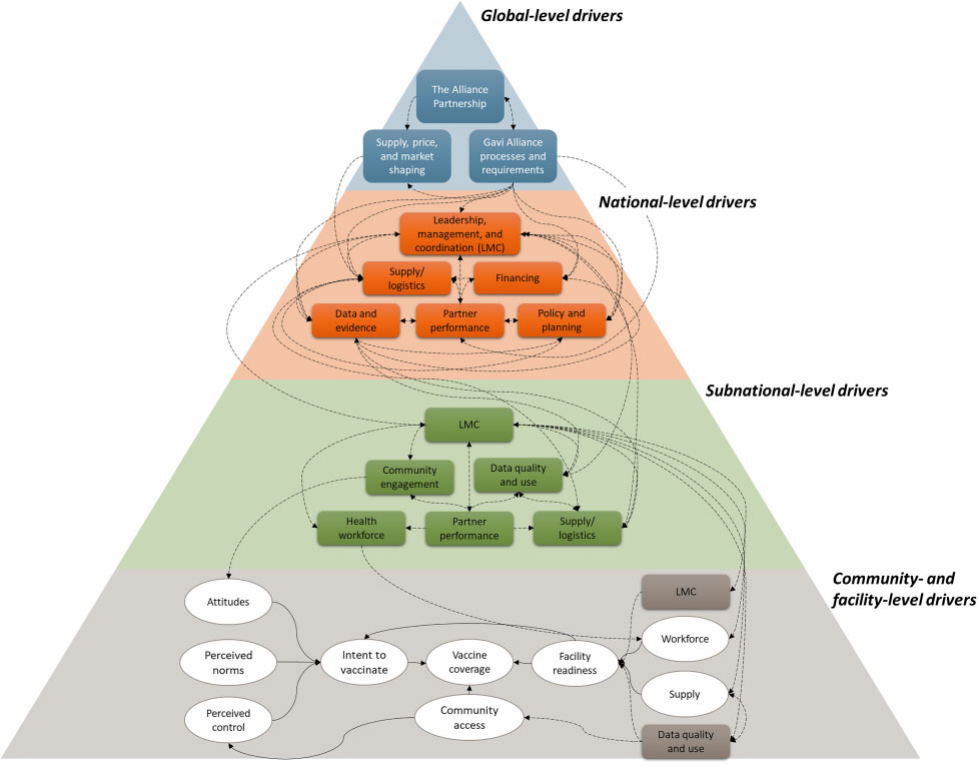
Revised conceptual framework of the drivers of vaccination coverage and equity

As Gavi’s strategy shifted to focus on improving coverage and equity through the levers of leadership, management and coordination, data, financing and supply chain, we responded by tailoring EQs to these emergent themes and activities. To comprehensively assess the drivers of coverage and equity, we developed a new conceptual framework that explicitly included a holistic range of drivers at all levels of the health system, in line with the goal of a conceptual framework to ‘bring together concepts to explain or predict a given event’ (Imenda, 2014). This framework (Figure 3) demonstrated our growing evidence base of the importance of district-level drivers of programme implementation and vaccine coverage, in particular district-level leadership and management. Each dimension pictured in Figure 3 has multiple sub-dimensions and was highly informed by frameworks from IR (Damschroder *et al.*, 2009; ExpandNet, 2019), as well as new conceptual frameworks from partners, the health systems building blocks, ecological models that consider health outcomes from an individual’s perspective and a systematic review produced as part of FCE (Phillips *et al.*, 2017).

Musing on *scope*: The scope of an evaluation of a complex programme should be broad enough to observe interdependencies between programme components; however, too broad a scope may limit the efficiency of the team, particularly at the outset. To produce actionable and relevant evidence, the scope of a prospective evaluation should be flexible.

### Methods, data and analysis

Table 3 describes the range of methods, data types, sources and tools. The main source of FCE data was through process tracking, namely meeting observation and key informant interviews (Table 3). At the outset, teams observed and tracked nearly all EPI activities at the national level and shifted increasingly to track implementation processes at sub-national levels, particularly around the launch of a new support stream (e.g. a measles campaign or human papillomavirus, HPV pilot project). Process tracking yielded primarily textual data, which we iteratively analysed and triangulated using qualitative techniques. We developed coding structures and qualitative codebooks based on each stream’s ToC and used a combination of pen-and-paper and digital software (NVivo 10 and Atlas.Ti 6) to organize data segments according to the ToC domains. As EQs became increasingly targeted and our focus shifted from descriptive to analytic, so did process tracking tools, employing structured checklists for meeting observations and evaluation rubrics to help analyse qualitative data.

**Table 3:** Summary of methods and data sources

Method	Stage of results chain	Purpose	Data sources	Example finding
Process tracking	All, except impact.	Monitored fidelity of programme activities and outputs against ToC to identify successes and challenges during programme implementation	Meeting observation of EPI technical working group and other key meetings, increasingly structured over time (e.g. using structured checklists) Document review of Gavi grant applications, budgets and performance frameworks (i.e. M&E frameworks), EPI policies, plans and strategies, published literature Vaccines doses administered from HMIS Fact checking interviews to clarify whether something occurred, to facilitate description of the process In-depth interviews to understand why and how the processes unfolded as observed	See the 2016 cross-country report for how years of process tracking was used to explain years of HSS grant implementation delays across FCE countries (Gavi Full Country Evaluations Team, 2017)
RCA	All, except impact.	Generates and tests hypotheses about the underlying causes of key programme success and challenges against all available evidence Synthesizes relevant evidence for developing key findings and recommendations	Desk review, key informant and fact-checking interviews, analysis of HMIS data	See Figure 4 for an example related to the sub-optimal coverage of new vaccines in Mozambique
Small-area estimates of vaccine coverage	Outcomes	Improve granularity of estimates of vaccine coverage in FCE countries to inform recommendations for resource targeting	Household surveys	Small-area estimates identified sub-national inequalities in each country
Observational study of determinants and constraints of effective vaccine coverage (Phillips *et al.*, 2018)	Outputs to outcomes	Identify determinants of effective vaccine coverage to inform recommendations for improvement	Household surveys with linked sero-surveys and health facility surveys Systematic review	See Phillips *et al*., 2018for estimates of the relative contribution of household and health systems determinants of the probability of a child being vaccinated
Resource tracking	Inputs	Estimate national resource envelopes and sources	Document review KIIs	Resource tracking results were reported in 2016 country reports and paved the way for further analyses of fiscal space and financing scenarios in 2017
Partnership/network analysis	Any (but primarily inputs to activities)	Evaluate the effectiveness, efficiency and country ownership of national immunization partnerships and their contribution programme performance	Social network surveys Document review In-depth interviews	We analysed the structure and composition of the Uganda HPV vaccine introduction partners’ network to explain successes and challenges observed during decision-making around HPV vaccine (Kamya *et al.*, 2016)
Vaccine effectiveness study	Impact	Estimate the impact of pneumococcal conjugate vaccine (PCV)	Nasopharyngeal carriage surveys Disease surveillance	Together, these studies provided strong evidence of the effectiveness and impact of PCV10 on nasopharyngeal carriage of vaccine-type pneumococcus and the incidence of vaccine-type invasive pneumococcal disease and pneumonia (Gavi Full Country Evaluations Team, 2017)
Regression analyses	Impact	Estimate the impact of new vaccine introductions of PCV and rotavirus vaccines on child mortality	Household surveys	Our analyses indicate that high coverage of new vaccines is associated with significant improvements in child mortality. In 2016, there were 10.1% (95% UI: 6.4, 13.8) and 11.9% (95% UI: 9.4, 14.3) reductions in under-5 mortality in Mozambique and Zambia, respectively, compared with scenarios where these vaccines were not introduced (Gavi Full Country Evaluations Team, 2017)
District case studies (Phase 2)	Inputs to activities to outputs to outcomes	Identify the drivers of district performance	HMIS data Document review In-depth interviews with district managers and health workers	District case studies identified barriers and enablers of vaccine coverage; see e.g. Uganda’s 2017–18 report (Gavi Full Country Evaluations Team, 2018)

Data from health management information systems (HMIS) on vaccine doses administered became an increasingly important data source for tracking implementation. In Phase 2, we used district-level data to select districts for district-level case studies. Initial attempts at integrating HMIS data into the process tracking framework suffered from infrequent access to systems and data, and slow analytic turn-around. In FCE Phase 2 we established stronger relationships with Ministry of Health (MoH) HMIS teams to enable more frequent data pulls directly into data visualization dashboards, allowing evaluation teams to identify unexpected results and explore their causes.

**Figure 4:**
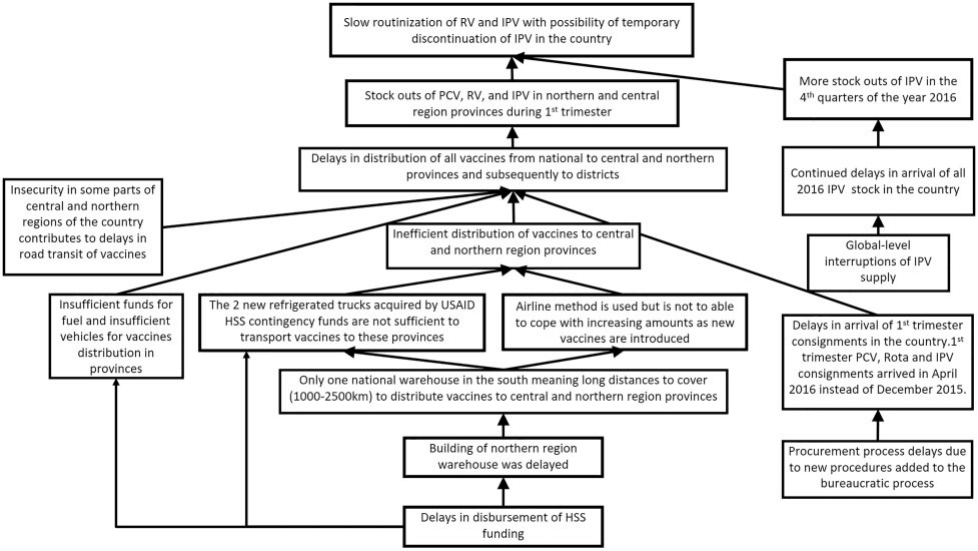
RCA diagram from Mozambique

When we observed a deviation from the expected process, e.g. a delay in the disbursement of funds from Gavi to the country, we used root cause analysis (RCA) to identify root causes of the observed challenge. Informed by theory, evidence and experience, we hypothesized likely root causes, then collected fit-for-purpose data, often from multiple sources, to test hypotheses. Team discussion and member checking with EPI stakeholders also contributed to our confidence in the accuracy of resulting RCA diagrams (see Figure 4 for an example). We iterated on RCAs as new lines of inquiry unearthed new evidence, and as the underlying system continued to respond and change.

The FCE implemented health facility surveys and household surveys, including sero-surveys in Uganda and Zambia. Household survey data were included in statistical models to estimate vaccine coverage at the 5 km^2^ level (‘small area estimates’), with the intention that we could use other quasi-experimental methods to measure the contribution of Gavi’s investments, whether in HSS or new vaccine introductions, on child mortality. For HSS, this was ultimately only possible in Bangladesh due to systemic delays in HSS implementation in the other countries.

In Phase 2 of the FCE we introduced comparative case studies of high and low-performing districts with the aim of identifying the drivers of differences in district performance, including effective implementation of the vaccine programme. In-depth interview questions with managers and health workers were informed by our revised conceptual framework and IR constructs (Damschroder *et al.*, 2009), with a focus on the role of district-level leadership and management capacity in the effectiveness of programme implementation.

Musings on *methods* and *data*: We recommend a wide range of methods and disciplines to enable a comprehensive evaluation of complex programmes, and concurrent mixing of methods and data types. These both require strong multidisciplinary teams. HMIS data allow more real-time feedback loops and bear additional attention and investment for evaluation and IR.

### Analysis and reporting

Evaluation teams were responsible for analysing their country-specific data across all EQs, with PATH responsible for analysing data collected at the global level. Cross-country synthesis occurred at annual in-person consortium meetings, where findings were discussed and compared. For example, we developed ‘mega RCAs’ which represented a middle-range theory for each observed challenge or evaluation priority (Pawson and Tilley, 1997). The 2016 report synthesized findings across countries and over the 2012–16 period (Gavi Full Country Evaluations Team, 2017). Table 4 presents examples of findings generated through multiple mixed methods for one case in Mozambique.

**Table 4:** Identifying the determinants of sub-optimal coverage of new vaccines in Mozambique

**Figure 5:**
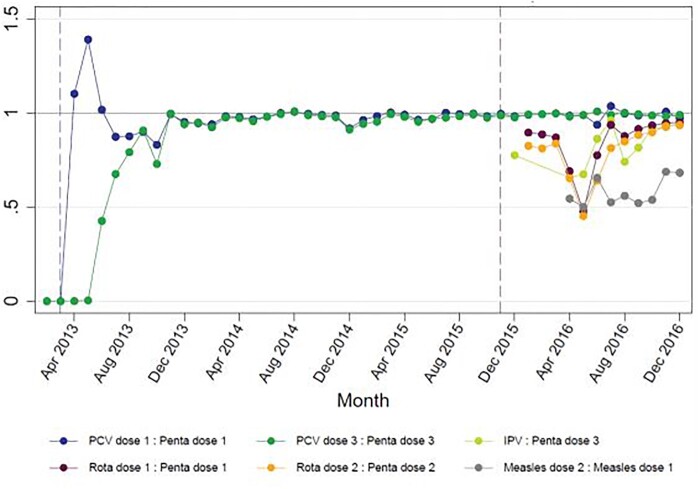
Ratio of number of doses administered of new vaccines compared with pentavalent vaccine, Mozambique

In 2015 Mozambique set out to introduce rotavirus vaccine, inactivated poliovirus vaccine (IPV) and MSD. The FCE team in Mozambique observed the planning for introduction closely, as the joint introduction of three vaccines was hypothesized to be a significant stretch of a relatively small national immunization programme. Through observation, document review and interviews, we concluded that the launch of these vaccines and their related activities (health worker trainings, communications activities, updating data systems) went smoothly in large part due to lessons learned from previous vaccine introductions (pentavalent in 2009; PCV in 2013) and high political will and commitment. However, using HMIS data, the FCE team observed that the number of doses administered of these new vaccines was lower than of the existing routine vaccines (Figure 5), prompting the development of a hypothesized RCA to identify potential root causes. Through meeting observation, interviews and document review, the team identified the following causes, illustrated in the final RCA (Figure 4): Late arrival of first-quarter vaccine consignments. Customs clearance challenges which protracted the procurement process. Lack of regional warehouses in the central and northern regions. Insufficient air transport capacity to deliver stock to central and northern regions. Global supply shortages of IPV. Because the FCE team was also continually tracking the process of implementing the country’s Health Systems Strengthening grant, they understood that many of the observed issues were caused by delays in receiving and implementing HSS funding from Gavi, which in turn had their own global and local root causes. The team’s ability to demonstrate the consequences of the HSS delays on vaccine coverage and suggest actionable recommendations led to changes in technical assistance in Mozambique and informed Gavi’s global reforms to HSS funding in 2016.

Based on feedback from stakeholders, we learned the importance of communicating both positive findings—what was working well—in addition to observed challenges or bottlenecks. Over time we also developed a ‘strength of findings’ rubric to communicate the level and strength of the evidence supporting the finding statement. In parallel, evaluators developed targeted and actionable recommendations based on emerging findings and through iteration: by revisiting data, deliberating and repeatedly refining statements. Formal recommendations were developed and documented on an annual basis as part of the FCE annual report and dissemination meeting in Phase 1, but in Phase 2 we co-developed recommendations with stakeholders which facilitated uptake and action. We also categorized recommendations thematically and in later years added a metric of urgency, categorizing recommendations as ‘act now’, ‘continue doing’ or ‘study further’.

### Dissemination and use of findings

Formal, planned disseminations included comprehensive annual reports and dissemination meetings, and in Phase 2 quarterly policy briefs. Gavi and national immunization programmes were asked to return a ‘management response’ indicating how they planned to act on each recommendation delivered in the formal reports. Informal dissemination of timely findings occurred in EPI meetings and through interpersonal relationships—these findings tended to be operational in nature and amenable to quick fixes by programme managers. Findings were used to inform Gavi and national decisions; we observed findings were more likely to be used when they were available in-time to inform a decision, leading to the introduction of the quarterly policy briefs and efforts to time evaluation activities around key decision-making windows (see the 2016 report for a section reporting use of findings) (Gavi Full Country Evaluations Team, 2017).

Musings on *analysis, reporting, dissemination, use of findings*: We recommend evaluators and their audiences continually discuss what evidence is needed for decision-making, and recognize that the value of additional evidence, and preferred type of evidence, will vary widely across stakeholders, countries and topics. Evaluators and funders should continually explore various dissemination strategies, which should be adequately resourced.

## Discussion

It is difficult to summarize the FCE approach, as it included so many combinations of methods, data and analytic approaches, across dozens of EQs. However, the more complex and adaptive it became, the more useful its findings were. The cost and complexity of the approach were significant compared with typical evaluations, but benefits included a deep understanding of the issues from daily engagement in them and rich contextual knowledge, strengthened capacity of local evaluators to undertake evaluation and IR of complex programmes, and institutional memory (and evidence) to sustain progress in FCE countries, and to buttress against swings in strategy at the global level. Costs can be lowered with larger proportion of in-country experts and leaner numbers of costly international and/and/or expertise from high-income countries.

The prospective approach of our study was well aligned with the constantly evolving environment of complex programmes, organizations and contexts. Its on-going nature provided a real-time feedback platform whose findings could be reported back to both Gavi and national immunization programmes and the results immediately used to modify programme processes so as to facilitate programme goals’ achievement. Specific examples of successes and failures of these real-time feedback loops are provided in Table 5. Bringing ‘what works’ to people who need it with greater speed and efficiency is one fundamental objective which IRs seek to address and this paper illustrates how a prospective evaluation is well suited to respond to such EQs.

**Table 5:** Strengths and weaknesses of the prospective evaluation approach and its influence on the implementation of Gavi support and EPI programmes

Successful example	Unsuccessful example	Opportunity for stronger evaluation design
Example 1: Feedback loops worked when individual, institutional and contextual factors aligned		
During year 1 of the evaluation in Mozambique, we found two major bottlenecks in the preparation for the introduction of PCV. First, the vaccine introduction grant funds were disbursed late by Gavi and only arrived in the country 2 weeks prior to the official launch of the vaccine resulting in lack of country readiness (e.g. the National Immunization Program did not have time to update immunization registers or prepare for adequate monitoring). Second, MoH and partners did not pilot social mobilization media messages resulting in the erroneous invitation of all infants rather than only the target age group (babies aged 4 months). Consequently, there was overuse of available doses and ensuing stock outs of PCV. On receiving these results both Gavi and Mozambique’s National Immunization Program made immediate process rectifications for the upcoming HPV vaccine introduction. Gavi disbursed funds 8 months prior to the launch and the National Immunization Program piloted HPV vaccine social media mobilization messages before widely implementing them.	Unfortunately, the findings from Mozambique’s PCV introduction in the first year of the FCE (e.g. that new vaccine introductions were sub-optimally planned) were repeated in almost every country for every new vaccine introduction, including in Mozambique for introductions in the years following the PCV introduction. The FCE evaluators continually recommended to Gavi that they play a more active role in determining introduction readiness, and specifically that countries and partner reflect on and propose adaptations based on previous new vaccine introduction experiences. The existence of these recommendations in reports, policy briefs, and in presentations were likely not married with adequate advocacy to Gavi or countries to ensure that they were sufficiently adopted.	Future prospective evaluations/IR platforms should invest more heavily in the use of evaluation findings for real-time and ongoing programme adjustments, which requires adequate resources for communications and advocacy, capacity building and institutionalization of a culture of learning and adaptation.

Example 2: Feedback loops were often too slow for larger policies or decisions		
A major focus of the evaluation was Gavi’s HPV vaccine ‘demonstration project’ policy—a policy that required countries to first demonstrate that they could achieve adequate coverage of HPV vaccine in select regions prior to applying for funding for national introduction. Evaluators found that Gavi’s policy incentivized countries to invest heavily in achieving high coverage rates in non-representative regions with greater demand and access to services; because of the incentive to demonstrate high coverage, learnings from these projects were of little relevance for national introduction and more challenging contexts. FCE evaluators communicated these and other findings to Gavi and over a period of multiple years and Gavi eventually reformed their policies and approaches to supporting decision-making, introduction and implementation of HPV vaccine based on FCE findings and other evidence.	It took Gavi multiple years to finalize the changes to their HPV vaccine policies and procedures, and in the meantime little action was taken both by Gavi and among partners to transfer cross-country lessons and adapt within countries. This contributed to sub-optimal implementation in countries that did decide to introduce (e.g. Uganda, Bangladesh) and delayed introductions in Zambia and Mozambique due to perceptions that lessons from the demonstration projects were not generalizable. These bottlenecks could have been avoided with faster and more adaptive engagement of Gavi and other Alliance partners.	Future prospective evaluations should experiment with approaches to supporting the timely adaptive management of programmes. This may mean more direct engagement with key decision-makers, or an agreement that learnings from the evaluation will be discussed and responded to by decision-makers

Example 3: Evaluation teams were not adequately embedded in programme decision-making		
Over time, evaluation teams strengthened their relationships and level of embeddedness with programme implementers. When evaluators were present during decision-making processes, they were able to add findings and evidence from the evaluation. This occurred in Bangladesh for their HSS-2 application (the evaluation team invited to participate on the technical working group), during Mozambique’s planning meetings for national HPV vaccine introduction, and frequently in Uganda where the team was the most embedded with the EPI programme.	In other cases, evaluators were not present for key decisions and that limited the incorporation of evidence from the FCE. In some cases—particularly in the early years of the evaluation—evaluators were present in decision-making meetings but felt unsure of whether and how to inform the decision without biasing it and calling into question their role as an independent evaluator.	Future prospective evaluations should encourage evaluators to be embedded in programmes.

Table 5 demonstrates that process evaluation alone was not sufficient to produce feedback loops and programme change. We believe this to be a combination of individual, institutional and contextual factors. At the individual level, evaluation teams should include skill sets (and funding) for communication, advocacy and policy translation. At the institutional level, evaluation funders should support and strengthen feedback loops by encouraging the embeddedness of evaluators and incentivizing learning and adaptation. Evaluation funders should remain open to reporting approaches that meet the needs of local users (e.g. conversations or short briefs) as opposed to long reports. Programmes and implementers should continue to take steps to improve individual and organizational capabilities to find and use evidence in decision-making. At the contextual level, evaluators, funders and implementers should reflect on which types of evaluation topics are most amenable to prospective evaluation and feedback loops. We found that decision-makers were more likely to be open to learning and adaptation for smaller operational decisions, and demonstrating successful feedback loops for these decisions might be a pathway to strengthen the overall culture of adaptation and learning.

Process evaluation was necessary to illuminate inside the black box of policy and programme implementation, and in the case of HSS investments, to describe the sub-optimal implementation of these investments. It is notable that while the FCE set out to evaluate the impact of HSS on vaccine coverage, too few HSS grant activities were implemented during the FCE to allow measurement, often at least partly due to Gavi rules or processes. Specifically for HSS, we recommend that any evaluation of HSS impact or return on investment be combined with process evaluation, and consider the full results chain from global and national decision-making to community and individual outcomes. Future retrospective evaluations of Gavi HSS support should leverage existing process evaluation finding from the FCE reports. When prospective process evaluation is not possible, retrospective evaluations of HSS should at least use mixed-methods and innovative analytic techniques that leverage both qualitative and quantitative data.

*Conflict of interest statement.* None declared.

*Ethical approval.* The Gavi Full Country Evaluations received ethical clearance from the University of Washington (USA), PATH (USA), icddr,b (Bangladesh), Universidade Eduardo Mondlane (Mozambique), Makarere University School of Medicine and Public Health (Uganda) and University of Zambia (Zambia).

## Supplementary Material

czaa127_Web_Appendix_1

## Footnotes

1 See www.gavi.org for all support streams, including disbursements by country since 2001.
