# Supplementary material for: How to evaluate the implementation of complex health programmes in low-income settings: the approach of the Gavi Full Country Evaluations
Source: Health Policy Plan. 2020 Nov 6;35(Suppl 2):ii35–46. doi: 10.1093/heapol/czaa127 (PMC7646739; doi:10.1093/heapol/czaa127)
Supplement: czaa127_Web_Appendix_1 [file czaa127_web_appendix_1.docx]

**Web Appendix 1. Evaluation questions over the lifetime of the Gavi Full Country Evaluations: Phases 1 and 2**

**Phase 1 questions from the original Request for Proposals**

- Inputs: to what extent are appropriate plans and funding in place?
- Processes: to what extent is implementation happening as planned?
- Outputs: to what extent is GAVI support to countries effective?
- Outcomes: to what extent are GAVI’s and countries’ goals being met?
- Impact: to what extent has population health impact occurred?

*Relevance*

- To what extent is the design of GAVI support and its implementation at country level aligned with GAVI priorities and principles?
- To what extent is the design of GAVI support and its implementation at country level relevant to the country’s needs, and aligned with the country’s priorities and systems?
- To what extent are the theory of change underpinning GAVI support to countries and GAVI’s fundamental policy assumptions valid?

*Effectiveness*

- To what extent does GAVI support to countries contribute to meeting the goals and objectives outlined in the 2011-2015 GAVI Alliance Strategy and business plan?  To what extent does GAVI support provided through each window of support meet the window’s objectives at country level?
- To what extent does GAVI support to countries contribute to helping countries meet the goals outlined in their national health strategies and plans?
- What is the added value of the GAVI Alliance as a partnership in contributing to results achieved at country level?
- To what extent is GAVI support to countries in line with aid effectiveness principles?

*Impact*

- What is the immunological evidence of effective vaccination?
- To what extent have reductions in morbidity and mortality of vaccine preventable diseases occurred?  To what extent has the GAVI Alliance contributed to such reductions?
- To what extent have reductions in child and adult mortality occurred in GAVI supported countries?  To what extent has the GAVI Alliance contributed to such reductions?
- To what extent has GAVI support contributed to social and financial risk protection for populations in countries supported by GAVI?
- To what extent does GAVI support contribute to improved equity between and within countries including, but not limited to, gender equity and equity between the poor and the non-poor?
- What positive or negative unintended consequences have occurred as a result of GAVI support to countries?

*Efficiency*

- To what extent is GAVI support cost-effective?
- To what extent have the following occurred in a timely manner: a) approval of cash support from GAVI, b) disbursement of money from GAVI to countries, c) utilisation of funds and implementation of activities by countries, and d) achievement of objectives?
- To what extent have the following occurred in a timely manner: a) approval of new and underused vaccine support from GAVI to countries, b) shipment and delivery of GAVI supported vaccines, c) utilisation of supply and implementation of immunisation programmes, and d) achievement of objectives?

*Sustainability*

- To what extent are the benefits of GAVI support to countries likely to continue after direct support has ended?

*Implementation*

- To what extent are GAVI’s policies and programmes coherent with countries’ national policies and programmes?
- To what extent is there evidence of transformative change? What are the key mechanisms or success factors that were most critical for transformative change?  Why and under what conditions did they come into effect?
- What are the most important factors that affect programme implementation, effectiveness, efficiency and sustainability?
- To what extent has GAVI support been responsive to changes in context?  In other words, to what extent have GAVI stakeholders used an adaptive management approach to learn from experience where appropriate?
- To what extent do the main stakeholders at country level contribute to the planning, implementation, monitoring and evaluation of GAVI support?  To what extent are their activities coherent and complementary?

**Additional questions added during Phase 1**

*Uganda*

- What is the quality of immunization services in the private health sector, compared to the public health sector, including health worker perspectives, in Uganda?

*Bangladesh Measles-Rubella campaign*

- What was the impact of the MR campaign on reducing susceptibility to measles and rubella in Bangladesh?
- What was the impact of the MR campaign on routine immunization systems in Bangladesh?

*Leadership, management, and coordination*

- What is the overall management burden associated with Gavi processes (especially HSS)?
- How does the implementation of the programmatic capacity assessment (PCA) in FCE countries add to or alleviate this burden?
- How will Gavi’s Programme Capacity Assessment contribute to strengthening country capacity to manage Gavi support?

*Health systems strengthening*

- To what extent does TA for HSS improve the application and implementation process?  To what extent and how does PEF change models of TA for HSS (e.g. more emphasis on in-country TA providers)?
- What are the country-level consequences of changes to HSS design (e.g. DFS)?
- Why is applying for Gavi HSS so challenging (e.g. time required, coordination, etc)?  To what extent are the challenges unique to Gavi, or common across HSS donors?
- To what extent does evidence drive the identification of bottlenecks for the HSS application, and HSS design align with major bottlenecks to immunization coverage and equity?
- To what extent is the design of HSS grants aligned with Gavi’s HSS principles, and with country priorities?
- To what extent does Gavi’s new IRC and approval process reduce the burden on countries to respond to clarifications, and enable the implementation process to begin in a more timely manner?
- Why is implementing Gavi HSS so challenging (e.g. time required, coordination, etc)?  To what extent are the challenges unique to Gavi, or common across HSS donors?

*Technical assistance*

- What TA models/approaches exist in FCE countries? Which are most perceived to be most effective?
- How has PEF changed the composition and structure of TA networks in-country, and how does this structure influence the ability to provide effective and efficient TA? To what extent does implementation of PEF shift the distribution of power and influence among partners?
- To what extent is the PEF-TCA process (e.g., JA, HLRP, PEF-MT) and the TA it funds perceived in 2016 to be building (or have the potential to build) capacity, to be increasing accountability, to be increasing transparency, and to be country-led.

*Sustainability*

- How are countries preparing for transition phase? What guidance and support is Gavi providing? What is the role of partners?
- How will Gavi’s Sustainability SFA strengthen the programmatic and financial sustainability of Gavi support?

*HPV vaccine*

- How have HPV demonstration projects influenced national introductions?

**Phase 2 questions**

1. What are the drivers of vaccine coverage and equity?

2. Whether, how, and why is Gavi support contributing to changes in vaccination coverage and equity?

3. What are the major factors influencing the achievement of these results?*

4. What has been the contribution of HSS funds to vaccine coverage in priority provinces and districts?

5. What are the advantages and consequences of managing HSS funds through partners, outside of government systems?

6. What is the effect of an interruption in Gavi HSS funding on routine service delivery, highlighting Government of Uganda and other partner funding?

7. Whether, why, and how is the introduction of measles, rubella (MR) vaccine in routine immunization being conducted as planned?

8. Whether, why, and how is the switch from pneumococcal conjugate vaccine (PCV) 10 to PCV13 being implemented as planned?

9. Whether, why, and how is an analysis of the lessons learned from previous support being taken into consideration?

10. Whether, why, and how is the human papillomavirus (HPV) national scale-up using the lessons learned from the HPV demonstration projects?

11. Whether, why, and how is the new HPV 2.0 policy facilitating national scale-up?

12. What are the demand-side reasons for the low coverage of HPV second dose in Uganda?

13. To what extent is the national introduction of HPV implemented as planned?

14. Whether, why, and how are country decisions to apply for new Gavi support taking into account the programmatic and financial sustainability aspects?

15. What are the drivers to increase financial support for immunization?

16. To what extent can recent programmatic gains of the Expanded Programme on Immunization (EPI) be sustained over time?

17. What are the positive and negative consequences of the new/updated Gavi processes?

18. What unintended positive and negative consequences occur as a result of Gavi support?

19. To what extent are the Gavi-supported activities that are designed to enhance performance management practices of the EPI effective in strengthening the Interagency Coordinating Committee and accountability across the program?

20. Why and how is the new Immunization Act affecting implementation (e.g., demand generation) and outcomes of Gavi support?

21. What is the composition of the immunization partnership in the country at national and district levels?

22. How effective is EPI management at the local level?
